# DETECT Schools Study Protocol: A Prospective Observational Cohort Surveillance Study Investigating the Impact of COVID-19 in Western Australian Schools

**DOI:** 10.3389/fpubh.2021.636921

**Published:** 2021-02-22

**Authors:** Marianne J. Mullane, Hannah M. Thomas, Melanie Epstein, Joelie Mandzufas, Narelle Mullan, Alexandra Whelan, Karen Lombardi, Tina Barrow, Sherlynn Ang, Adele Leahy, Ewan Cameron, Leanne Lester, Matt Cooper, Paul Stevenson, Mitch Hartman, Anne McKenzie, Francis Mitrou, Stephen R. Zubrick, Juli Coffin, Donna Cross, Asha C. Bowen, Peter Gething

**Affiliations:** ^1^Telethon Kids Institute, Perth, WA, Australia; ^2^Cancer Council Western Australia, Perth, WA, Australia; ^3^Edith Cowan University, Perth, WA, Australia; ^4^Curtin University, Perth, WA, Australia; ^5^University of Western Australia, Perth, WA, Australia; ^6^Child and Adolescent Health Service, Perth, WA, Australia

**Keywords:** COVID-19, school, child health, screening, wellbeing, mental health

## Abstract

**Introduction:** Amidst the evolving COVID-19 pandemic, understanding the transmission dynamics of the SARS-CoV-2 virus is key to providing peace of mind for the community and informing policy-making decisions. While available data suggest that school-aged children are not significant spreaders of SARS-CoV-2, the possibility of transmission in schools remains an ongoing concern, especially among an aging teaching workforce. Even in low-prevalence settings, communities must balance the potential risk of transmission with the need for students' ongoing education. Through the roll out of high-throughput school-based SARS-CoV-2 testing, enhanced follow-up for individuals exposed to COVID-19 and wellbeing surveys, this study investigates the dynamics of SARS-CoV-2 transmission and the current psychosocial wellbeing impacts of the pandemic in school communities.

**Methods:** The DETECT Schools Study is a prospective observational cohort surveillance study in 79 schools across Western Australia (WA), Australia. To investigate the incidence, transmission and impact of SARS-CoV-2 in schools, the study comprises three “modules”: Module 1) Spot-testing in schools to screen for asymptomatic SARS-CoV-2; Module 2) Enhanced surveillance of close contacts following the identification of any COVID-19 case to determine the secondary attack rate of SARS-CoV-2 in a school setting; and Module 3) Survey monitoring of school staff, students and their parents to assess psycho-social wellbeing following the first wave of the COVID-19 pandemic in WA.

**Clinical Trial Registration:** Trial registration number: ACTRN12620000922976

## Introduction

The first cases of COVID-19, a novel infection caused by the virus now designated SARS-CoV-2, were notified in December 2019 (1, 2). The subsequent global COVID-19 pandemic has elicited an unprecedented public health response, with many governments implementing disease control measures that most people have never previously experienced in their lifetime. Commonly implemented control measures include physical distancing, restrictions on public and private gatherings, closure of workplaces and hospitality venues, travel bans, and school closures (3). Three quarters of schools globally have experienced closures in 2020 (4). Across Australia, and indeed globally, whether schools are sufficiently safe and should remain open for staff and students remains a contentious and widely discussed topic. While it is important to stop the spread of COVID-19, predictions of increased economic, social, and psychological burden on families lead some to debate the wisdom of removing children from schools (5–7).

COVID-19 prevalence in Australia has been low relative to the global context, with just over 27,500 cases reported to October 30, 2020. The state of Western Australia (WA), with a population of ~2.5 million, has experienced only 775 cases, 9 deaths and no confirmed community transmission since April 2020. After a period of low school attendance and school holidays (March 15–April 29), during which parents were encouraged to provide home learning to their children, all students in WA were required to return to school from April 29, 2020. This call to return students to school sparked community concern about student and school staff safety. While existing data indicates the COVID-19 burden and rate of transmission in children and young people is much lower than adults (8–10), there is still much to be learnt about the transmission dynamics of SARS-CoV-2 in these younger age groups.

In addition to gaining an understanding of SARS-CoV-2 transmission in children and young people, the influence of social distancing measures such as school closures on their psychological health and wellbeing needs to be explored (11). Evidence suggests that children and adolescents have experienced increased levels of anxiety, irritability and inattention due to school closures (12), and that their online behaviors have been impacted, with young people engaging in more screen time for both educational and social purposes (13). Connectedness with friends and school can influence children and adolescents' life satisfaction, though to date it is unclear how these factors have mediated the influence of COVID-19.

In late April 2020, the WA government instigated the DETECT Schools Study in partnership with the Telethon Kids Institute to assess the prevalence and impact of COVID-19 in WA schools. The study, detailed here, seeks to quantify and characterize the transmission of SARS-CoV-2 in WA school settings while investigating the wellbeing of students, teachers, and parents during the pandemic.

## Study Design

The DETECT Schools Study is a prospective observational cohort surveillance study currently underway in 79 WA government schools, including education support settings and residential colleges. These schools are distributed across our large state, over an area of 2.6 million km^2^. Data collection will initially be conducted for 6 months and may be extended beyond this depending on the evolving COVID-19 situation.

### Aim and Objectives

The overall aim of the DETECT Schools Study is to better understand the burden, transmission, and psycho-social factors associated with COVID-19 in WA school communities. To this end, the study comprises three modules with distinct objectives:

- Module 1 (asymptomatic SARS-CoV-2 testing): to investigate the prevalence of asymptomatic SARS-CoV-2 infection in a cohort of randomly selected school students and staff (in 40/79 participating schools).
- Module 2 (SARS-CoV-2 index case response): to determine the secondary attack rate of any SARS-CoV-2 infections detected in a WA school setting (in 40/79 participating schools).
- Module 3 (wellbeing factors associated with the COVID-19 pandemic): to elicit self-reported data on the physical, social and emotional wellbeing of students, their parents, and staff (in all 79 participating schools).

Modules 1 and 3 of the study are in progress, and Module 2 will be initiated as needed.

### Stakeholders/Partnerships

The DETECT Schools Study is a WA Government initiative, coordinated by the Telethon Kids Institute in partnership and consultation with the WA Departments of Health and Education. Study design, operational oversight and data interpretation is led by the Telethon Kids Institute study team. School selection and management is coordinated by the Department of Education. Swab testing is coordinated by the Department of Health: samples are collected by the Child and Adolescent Health Service (CAHS) and the WA Country Health Service (WACHS), and SARS-CoV-2 testing is performed by public laboratory service provider PathWest.

### Patient and Public Involvement

Community involvement and advice was actively sought in the design and preparation of this study and will inform the analysis and dissemination of findings. A National Community Advisory Group for COVID-19 Research, convened by the Telethon Kids Institute, reviewed and provided feedback on procedures and resources including consent forms and surveys to aid in the development of content most appropriate for potential study participants, including children with disabilities. This panel comprised community members from across Australia, including Aboriginal members. Telethon Kids Institute Kulunga Aboriginal Research Development Unit provided advice regarding communications with Aboriginal families who may be involved in the study, including culturally-secure and informed consent processes and measures for supporting Aboriginal families during Module 2, which requires swabbing and symptom checking to take place within the home. Further, advice was sought from the Department of Education and the four participating education support schools to develop visual communication tools to increase accessibility of the survey for students in these settings.

While considerable time was spent refining the DETECT Schools Study protocol and resources, the study proceeds with an acute awareness that implementation may well bring to light additional considerations or challenges. As such, consultation with these advisement bodies is ongoing and will continue to be critical in informing the trial as it progresses.

### Training of Swabbing Staff

Testing team members are trained in swab collection and management by a pediatric infectious disease specialist (AB). This training includes online and/or face-to-face training sessions, and access to swab collection training videos and Standard Operating Procedures for swab collection and transport. Testing team members are also trained in the use of the REDCap database for data collection during in-school swabbing. REDCap training and a School Coordinator Guide are also provided to a School Coordinator from each participating school to aid in survey data collection.

### Study Methods

#### School Selection and Group Assignment

Eighty schools were selected for participation by the Department of Education, ensuring the inclusion of broad representation across state geography, primary and secondary schools, and specialist facilities including residential colleges and education support settings. A variety of practical considerations with respect to maximizing school participation and project feasibility were considered. Consequently, school selection did not follow a strict randomization procedure from all schools state-wide, although care was taken to minimize biased selection based on any *a priori* criteria. The potential for bias due to this non-random selection will be addressed during data analyses, where sample weightings will be employed to adjust for under- or over-representation across the geographical and demographic characteristics of each school, as determined from Australian Bureau of Statistics (ABS) census data.

The eighty participating schools were stratified into 10 groups by geographical region and school classification (Table 1).

**Table 1:** Stratification of DETECT Schools by geographical region and school type.

	**North metro**	**South metro**	**Regional**
Primary	12	12	12
Secondary	12	12	12
Educational support setting	2	2	–
Residential college	1	3	

Within each stratum, schools were allocated to either the swab testing or non-swab testing groups by a simple randomization procedure. Forty schools were randomly allocated to the swab testing group and were invited to participate in Modules 1 (asymptomatic SARS-CoV-2 testing), 2 (SARS-CoV-2 index case response) and 3 (wellbeing survey). The remaining 40 schools were randomly assigned to the non-swab testing group and were invited to participate in Module 3 only. Following recruitment and randomization one non-swab testing metropolitan secondary school opted out of the study, leaving 79 participating schools.

#### Consent Process

Distinct consent was sought for each study module. Invited participants consented to the three study modules (swab testing group) or Module 3 only (non-swab testing group) at the beginning of the study through one online consent process supported by the REDCap platform (14). Participation in each module is stand-alone, and individuals can choose not to participate in any module without affecting their participation in other modules.

Modules 1 and 2 require active consent from participants, collected from staff and students' parents/guardians at the 40 swab testing schools. Consenting individuals (or guardians) complete a consent form in the study database and may subsequently withdraw at any time.

For Module 3, a passive consent approach is adopted for students: parents are required to actively “opt-out” their child from participating and the survey is offered to all students who have not opted out. Previously, application of an opt-out consent framework nationally by the Australian Early Development Census (AEDC) resulted in a minimum participation rate of 95%, serving to minimize selection bias and maximize the reliability and generalisability of the research data collected. The use of student passive consent for this module was supported by the consumer reference group and ethics committees. Parents and staff provide active consent to participate but may opt out of participation at any time.

#### Module 1 Methodology

Module 1 entails combination throat and nose SARS-CoV-2 swabbing of asymptomatic students and staff to quantify how much (if any) asymptomatic infection is present in schools. Students at school on the day of testing are assumed to be asymptomatic, as the Department of Education has adopted a policy supporting families to keep any children with COVID-19 symptoms at home. From the consented population, up to 150 randomly selected students and staff are swabbed in each school per round, with staff comprising 10% of the sample.

##### Exclusion Criteria

Participants are excluded from swabbing if they have experienced any of the following:

- More than one nosebleed per week.
- Recurrent vomiting, particularly if triggered by a gag reflex.
- Large/problematic tonsils awaiting tonsillectomy.
- Significant anxiety about medical procedures.

During the consent process, parents were asked to confirm that their child(ren) did not meet the exclusion criteria. Staff were asked to self-assess against the exclusion criteria.

##### Randomization for Swab Testing

Three to six rounds of swabbing will be conducted, approximately monthly. Of students and staff who provided active consent, 150 will be randomly selected from each school in each round. Student grade level groups will be stratified into two strata per school (younger and older halves of students, depending on the total age demographic of the school). Randomization will be conducted within the REDCap database using a simple random number algorithm, generating a list of 200 consenting participants: 45% students from each stratum and 10% staff. If a randomized student or staff member is not available or does not assent to testing on the day, they will be replaced by the next available consenting participant from their stratum until 150 swabs are collected.

##### SARS-CoV-2 Swabbing

**Figure 1:**
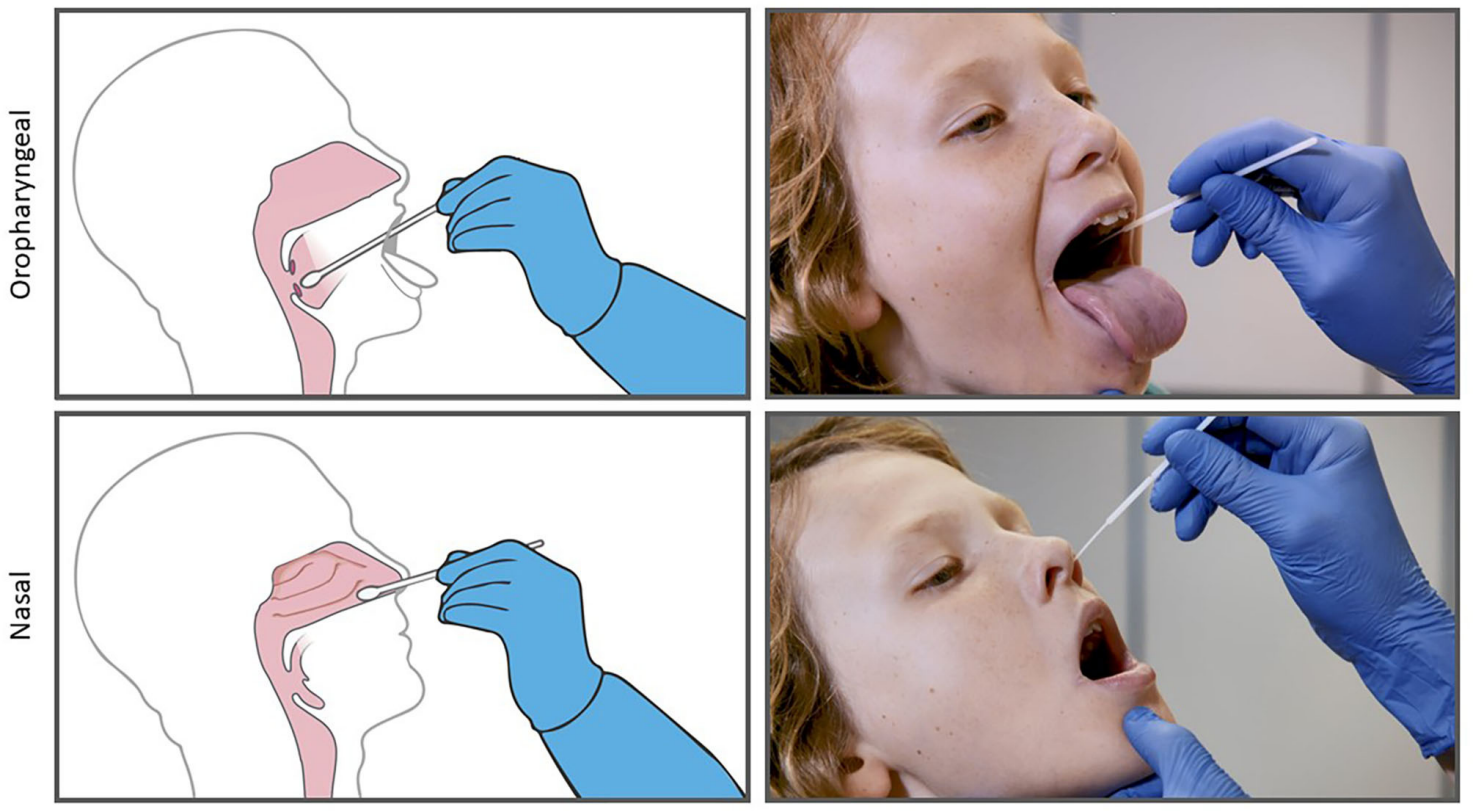
Combined oropharyngeal and nasal (OP/Na) swabbing with a single fine flexible shaft flocked swab.

SARS-CoV-2 swabbing will be carried out by a team of nursing staff. The participant's information will be verified to ensure consent has been obtained, and if for any reason identification and therefore consent cannot be confirmed (e.g., student indicates a date of birth different to that captured during the consent process), then swabbing will not continue with that individual. All participants will be asked to verbally assent prior to swabbing being performed. An oropharyngeal (OP) swab (tonsillar beds and the back of the throat, avoiding the tongue) and subsequent nasal (Na) swab (along the floor of the nasal cavity parallel to the palate to a depth of 1-2 cm before resistance is encountered at the nasal turbinates) (Figure 1) will be taken sequentially with a single fine flexible shaft flocked swab (CITOSWAB Flocked Swab, Gaia Science, Singapore) and placed into virus transport media (VTM). If at any point the participant indicates they would like to stop or displays signs of distress, swabbing will not proceed.

The combined oropharyngeal and anterior nasal (OP/Na) swab employed for this study is not the same as the nasopharyngeal swab commonly used for clinical SARS-CoV-2 diagnosis. OP/Na swabbing is significantly less invasive and therefore less likely to cause discomfort. This allows for the swabbing of students without a parent present, minimizes discomfort and optimizes the rate of assent amongst participants. Recent research indicates that OP/Na swabbing has comparable specificity and sensitivity to nasopharyngeal swabbing for SARS-CoV-2 detection (15). This swabbing approach aligns with the current recommendations from the Communicable Diseases Network Australia (CDNA) COVID-19 National Guidelines for Public Health Units (version 3.6, 30 July 2020), which states that for most patients with mild illness (this is the closest to asymptomatic), collection of OP/Na swabs is a low risk procedue that can be performed with appropriate contact and droplet precautions. Testing staff will wear an apron, mask and eye shield at all times when testing, and a new pair of disposable gloves for each participant.

##### Laboratory Analysis of Swabs

Polymerase chain reaction (PCR) testing of all swabs for SARS-CoV-2 will be carried out at PathWest using an in-house PCR platform of high analytical sensitivity and specificity with appropriate positive and negative controls. To reduce the chance of a false positive result, any sample which returns a positive result will undergo confirmatory testing with the GeneXpert SARS-CoV-2 PCR platform (Cepheid, California, USA). Testing of asymptomatic participants has the greatest risk of returning an unexpected false positive, and use of a second platform with robust internal controls will minimize the risk of reporting a false positive result.

##### Notification of Swab Results

Under the Public Health Act 2016 of WA, COVID-19 is a notifiable disease. Any negative SARS-CoV-2 results will be communicated to participants directly by text message from PathWest, in line with standard clinical care notification procedures. In the instance of a positive SARS-CoV-2 result, PathWest will notify the WA Health Communicable Diseases unit, who will initiate standard contact tracing procedures with the confirmed case and liaise with the key contact for the relevant school to ensure appropriate public health action. The Department of Education recommends closure and deep cleaning of any school in which an index case presents, and this will be actioned by the Department of Health. At all times, the identity of the positive case will be kept confidential.

For study data analysis, all SARS-CoV-2 results will be communicated to the study team by means of a secure and confidential file transfer.

##### Swab Testing Sample Size Calculation

The study sample size (40 schools, 150 sampled individuals at each school per month, for 3–6 months) is calculated with intent to achieve as broad and representative a sampling frame as possible. Taking into account practical considerations such as constraints on school participation and feasible total number of samples per month, and given the potential sources of bias and imprecision inherent with these constraints and the likely very low prevalence rate, the task of estimating state-wide infection prevalence among school populations does not lend itself to simple statistical analysis. As such, this study will employ a sophisticated approach validated using simulated data, which explores its performance in a range of different community transmission intensity scenarios.

##### Data Analysis

This study seeks to design a robust yet efficient estimator for the prevalence of SARS-CoV-2 infections amongst the general population of school children and staff in WA based on Module 1 testing data. The challenge will be to bound the range of plausible sampling distributions that might arise from observation through the given survey design of the epidemic spread of this virus, especially given the many uncertainties that remain concerning SARS-CoV-2 transmission dynamics.

Under these uncertain epidemic conditions, standard approaches to prevalence estimation based on adjustment by estimated design effects for representing intra-cluster correlation may perform unfavorably. A solution for this unique situation is to adopt an estimator designed specifically for robustness against model misspecification. Analysis of Module 1 data will employ a weighted likelihood bootstrap with Bayesian regularization. This nominal model supposes a Poisson sampling distribution with a Gamma distribution prior on the unknown rate equivalent to a single prior observation of one positive case amongst 6000 (40x150) individuals.

#### Module 2 Methodology

Module 2 entails enhanced surveillance of the close contacts of any COVID-19 index case identified in a DETECT swab testing school, with a view to identifying any onward transmission and thus elucidating the role that schools may play in the transmissibility of SARS-CoV-2 between students and staff. In tandem to routine public health surveillance, Module 2 activities will include further swabbing and monitoring of students and staff consistent with existing methodologies such as the World Health Organization's First Few Hundred (FFX) Cases Methodology (16). Module 2 will be activated in response to an index case in any swab testing school, regardless of whether detection was through Module 1 or community swabbing.

##### Identification of Close Contacts

In the event of a positive SARS-CoV-2 result, the WA Health Communicable Diseases unit will be informed. Routine public health follow-up by this unit will begin immediately, including contact tracing to identify close contacts of the case. A close contact is defined as anyone with >15 min of face-to-face contact or >2 h in the same room with a confirmed case.

A list of all individuals at the school who have consented to Module 2 participation will be merged with the Public Health unit's close contact list by secure file transfer, identifying all close contacts of the case who have consented to participation in Module 2.

##### Enhanced Follow-Up Activities

###### Swabbing

Study investigators will telephone each consented close contact to confirm their participation in Module 2, emphasizing the distinction between routine (compulsory) public health instruction and voluntary participation in the study. Sample collectors will attend the participant's residence to collect an OP/Na swab twice during their 14-day isolation period–between days 4–8 and days 11–14–regardless of whether the participant is symptomatic or asymptomatic. Swabs will be processed, and results conveyed to participants, as described for Module 1. Any positive results will be immediately notified to the WA Health Communicable Diseases unit.

###### Symptom Checking

Routine public health surveillance requires COVID-19 close contacts to self-monitor for symptoms during their 14-day isolation. Close contacts recruited to Module 2 will also be invited by SMS to complete a daily online symptom diary on days 4–14 of isolation, monitoring the participant's health and for symptoms of COVID-19. Participants who indicate symptoms consistent with the COVID-19 case definition [fever ≥38°C or history of fever (e.g., night sweats, chills) or acute respiratory infection (e.g., cough, shortness of breath, and sore throat)] will be advised to notify the WA Health Communicable Diseases unit immediately.

###### Serology Testing

Module 2 participants, regardless of whether they experience COVID-19 symptoms, will be offered a serology test (testing a blood sample for antibodies which indicate a previous SARS-CoV-2 infection) 1 month after their exposure to the index case. If participants give consent for this test, they will attend a PathWest collection center for serology testing between days 28–42 post-exposure. Blood samples will be centrifuged within 24 hours of collection, and detection of antibodies (IgG and IA) carried out by PathWest using the EUROIMMUN serological assay (17). Participants may decline the serology test while still taking part in other Module 2 surveillance activities, without impacting their ongoing care. As serology testing is not part of the standard public health follow-up of COVID-19 close contacts, notification of results will be completed by the study team rather than the public health unit.

#### Module 3 Methodology

Module 3 is a quantitative, quasi-experimental, cross-sectional pulse survey administered in all 79 study schools immediately following the first round of swab testing, with follow-up in Term 4, 2020. It is designed to identify psycho-social wellbeing factors associated with the COVID-19 pandemic among school-aged children, their parents and school staff in WA. Specifically, the survey aims to investigate:

1. How COVID-19 public health interventions are differentially associated with students' and their parents' and teachers' physical, social, and emotional wellbeing across sub-populations (e.g., age, school system) and geography.
2. How COVID-19 social interventions are differentially associated with students' learning and schools' operations across sub-populations (e.g., grade level group, school type) and geography.
3. How students, school staff and parents in swab testing group schools (40/79 participating schools) felt about the COVID-19 screening activities of Module 1.

##### Participation

All 79 DETECT study schools will participate in Module 3. Surveys will be administered to all students aged 9–18 years (Grades 4–12) who have not been opted out, and parents of children aged 3–18 years (Grades Kindergarten-12) and school staff who actively consented to participate. As participation requires completion of a self-report survey in English, participating schools have been consulted to understand the cultural diversity of their community and where possible support will be provided to allow those who do not speak English as a first language to participate. Parents who do not have internet access will not be able to complete the survey, and as such are not included in the study, although provision will be made for paper consent. Some sample loss is inevitable due to individuals choosing not to participate, however measures taken to promote participation include:

1. Targeted and informative communications to parents of eligible children and school staff, explaining the value of participation.
2. Development of separate surveys for different age groups with regard for developmental capabilities and educational settings.
3. Ensuring all responses are anonymous and confidential, with no data to be published in a way that could risk identification of an individual or school.

In total, five survey instruments were designed specifically for this study: for primary school students (Grades 4-6); high school students (Grades 7-12); school staff; parents of primary school children (Kindergarten-Grade 6); and parents of high school children (Grades 7-12). For pragmatic and budgetary reasons, surveying children below Grade 4 was outside the scope of this study. All survey instruments contain a set of Hogben questions allowing for the linkage of baseline and follow-up responses while maintaining anonymity (18).

Surveys utilize validated scales including the Child Health Utility Index (CHU9D) (19) and the WHO (Five) wellbeing Index (1998 version) (20), allowing for future comparison of results with Australian pre-COVID-19 benchmark data. Other scales are adapted for the survey, including the Students' Life Satisfaction Scale (SLSS) (21); and individual items are adapted and/or used in isolation from the CoRonavIruS Health Impact Survey (CRISIS) V0.3 (22); the COVID-19 Adolescent Symptom & Psychological Experience Questionnaire (CASPE) (23); Measuring Worldwide COVID-19 Attitudes and Beliefs (24); the National Survey of Child and Adolescent Mental Health and Wellbeing 2012 (25, 26); and the Speaking Out Survey (2019) (27). Supplementary Table 1 provides all tools and items included in each survey instrument.

##### Survey Data Collection

Wellbeing surveys will be administered to participants online using REDCap survey software (14). Email addresses, obtained from participating school staff and parents through the consent process, will be used to distribute anonymous, untracked school-specific survey links. Students will complete surveys in class, organized in each school by a trained School Coordinator who will follow a standardized survey administration protocol (outlined in the School Coordinator Guide). This guide includes instructions for survey completion, including the provision of a mental health fact sheet to all participating students and implementation of specific procedures if a student shows signs of distress.

The first cycle of survey collection (Phase 1) will be conducted following the completion of the first round of Module 1 swabbing. A second cycle of surveys (Phase 2) will be completed before schools close for the year. Given the dynamic nature of schools' responses to state requirements, COVID-19 prevalence, and broad COVID-19 social policy, differential timing of survey administration has the potential to confound results. To address this, schools are “matched” (one “swab testing” school with one “non-swab testing” school) according to school size, type and geography. Matched schools will complete the survey at the same time.

##### Survey Data Analysis

Purposeful selection of participating schools by the Department of Education may dispose the study to bias. To mitigate problems that may arise in generalization, participating schools in both conditions will be pooled and compared to the WA Department of Education estimates based on all WA Government Schools. Comparisons will be made with respect to student composition, school size, and National Assessment Program-Literacy and Numeracy (NAPLAN) performances.

Without identification of individual schools, aggregated Phase 1 results will be analyzed using descriptive statistics, including frequencies and prevalence estimates, to determine whether any differences in demographics (survey condition, parent gender, parent age, language other than English spoken at home, Aboriginal or Torres Strait Islander, family type, number of children in family, child disability, child gender, EA school, and school region) exist for child and parent wellbeing, resources for children learning at home during COVID-19, experience of schools handling COVID-19, learning choices during COVID-19, or involvement in the DETECT study.

Multivariate multi-level (linear, multinomial, and logistic) models will be employed to determine which factors may be associated with changes in key outcome measures, including demographic predictors (parent gender; parent age; LOTE; ATSI; family type; number of children; child gender; child year level; child disability, EAS school, and school region) of child and parent wellbeing, parent self-efficacy, parent or staff positive and negative emotions, parent or staff stress, total amount of resources for home learning, positive experience of schools handling COVID-19, testing concerns, learning choices, reasons for keeping children at home, and reasons for sending children to school. These models will account for the clustered nature of the data and control for school-related variables such as school size and socioeconomic status (SES).

Phase 2 results will be analyzed in the same manner. In addition, comparisons will be made to identify any significant differences between Phase 1 and Phase 2 wellbeing survey results and between swab testing and non-swab testing schools.

##### Data Confidentiality

Raw data will be securely stored in REDCap on a password protected server, to which only researchers associated with this project have access. Data will be encrypted prior to distribution between investigators and shared through secure online portals. Each collection's data (each school and each student/parent/staff within a school) will be stored in different areas of the database, with segregation of identifying data where possible. Analyses will only be conducted on de-identified datasets.

##### Reporting and Dissemination of Data

This study is designed to inform policy and practice in public health, disease screening and education settings. Interim (post-Phase 1 survey collection) and final (post-Phase 2 survey collection) reports will be delivered to Government stakeholders outlining the findings of the study. A series of academic publications will be published, focussed on key findings and subsets of the data collected. While schools will remain anonymous in reporting and publication, findings from the wellbeing surveys will be used to inform school- and community-based intervention as necessary: as such, school-specific results will be shared with each participating school to inform policy and practice.

## Discussion

The COVID-19 pandemic has had high rates of infectivity (28), serious health impacts for many (29) and wide-spread influences on socio-economic factors, with lasting impacts predicted on both physical health and mental wellbeing (30). Dynamic decisions are being made at individual and policy levels about the safety of social interactions, including attendance at workplaces and schools (31, 32), and throughout the pandemic almost 75% of schools globally have experienced some magnitude of closure (4). This response was based primarily on a large body of existing data which implicates school-aged children as major spreaders of influenza (33). It is now understood that this may not be the case for SARS-CoV-2 (34–36), and that the incidence of transmission from child to child or child to adult appears to be low (37, 38).

With SARS-CoV-2 now a widespread respiratory virus, we must learn how to live with COVID-19 in a way which allows for children's safe and continued education (39)–while transmission events within schools remain a risk, the greater risk to children is arguably in missing out on the educational and broader social benefits of school attendance. To inform this balancing act in WA, two pieces of information are key: firstly, what is the likelihood of SARS-CoV-2 transmission in schools? Secondly, what are the ongoing social and emotional impacts associated with COVID-19 on school communities?

With parents instructed to send their children to school if they are well, Module 1 of this study is designed to detect asymptomatic SARS-CoV-2 infection in schools. Other studies have investigated asymptomatic SARS-CoV-2 incidence in pediatric settings, and found low prevalence among those attending pre- and primary schools in the UK (0.004% in students, 0.01% in staff) (40), American children presenting to hospital for surgical care (0.65%) (41) and Italian children presenting to a pediatric emergency department (1.2%) (42). By screening and thus increasing the likelihood that asymptomatic SARS-CoV-2 infection will be detected, we aim to determine if asymptomatic SARS-CoV-2 infection rates are similarly low in WA schools; providing advice to the community and adding to the evidence base drawn on for state government policy making.

The DETECT Schools approach to SARS-CoV-2 swabbing diverges from the nasopharyngeal sampling commonly used for COVID-19 testing in clinics. Nasopharyngeal swabbing is uncomfortable, leading to a high rate of non-compliance, and emerging data suggests that various non-nasopharyngeal samples are appropriate for the detection of SARS-CoV-2 (43, 44). Data suggest that both saliva (45) and nasal (46) swabs can be used for virus detection, and the DETECT Schools Study sampling method combines the collection of nasal and oral samples to maximize the sensitivity of testing while causing minimal discomfort. This approach has recently been employed in some healthcare settings for pediatric testing (47, 48), and significantly increases the tolerability of testing. If accepted by the pediatric DETECT cohort, this validated testing approach could be repurposed in the event of increased community transmission in WA for rapid COVID-19 screening in other settings including universities, childcare and aged care facilities.

Macartney et al. have reported that students and staff who attended schools in New South Wales (NSW), Australia while infectious with SARS-CoV-2 did not contribute significantly to COVID-19 transmission through their school attendance (49). Module 2 of this study aims to similarly investigate the secondary attack rate of SARS-CoV-2 in WA schools, and inform understandings of virus transmission generally. In addition, participants who find themselves close contacts of a COVID-19 case will benefit from more comprehensive monitoring than is provided by standard public health follow-up, enhancing the likelihood of early detection and treatment if infected. This complements a First Few Hundred (FFX)-based process currently being orchestrated by WA Health which focuses on within-household transmission. The DETECT Module 2 extension to include schools in this enhanced follow-up will not only provide crucial information to inform the risk of transmission within schools, but also contribute to the overall public health response within WA.

While much remains to be learnt about the transmission dynamics of this new virus, the impact of the pandemic on the population's psychosocial wellbeing is also not well understood (50). Much of the current research focusses on this question in areas of high prevalence, where communities have been exposed to extensive lockdowns and other disruptive changes which could have an immediate and lasting impact on social, physical, and emotional wellbeing (51, 52). WA, in contrast, has experienced a low incidence of COVID-19 cases to date. Hence, Module 3 of this study will provide an opportunity to examine how the pandemic has impacted the wellbeing of students, parents and staff in this low prevalence setting.

WA children have witnessed the wide-spread impact of COVID-19. They have also experienced unprecedented disease prevention measures including optional school attendance and unpredictable changes in schooling, parental employment, and income and housing security. Gaining an understanding of the impact this may have had on their wellbeing will help to guide nimble local and systemic psychosocial policy and intervention, particularly regarding the maintenance of school attendance. This insight may well be applicable to other low prevalence settings and will be complementary to other contrasting research which focusses on the epidemiological and psychosocial implications of COVID-19 in areas of high prevalence. Further to this, the complementary roll-out of Module 1 and Module 3 activities will allow for assessment of the impact that school-based screening has on community anxiety, informing thoughtful approaches for future track and trace endeavors.

Across the globe, cross-disciplinary partnerships have proven vital in society's rapid response to the COVID-19 pandemic (53). In an Australian context, the DETECT Schools Study illustrates a framework for the bringing together of government, health service providers, researchers, and the community to rapidly roll out minimally invasive and efficient testing and psychosocial tracking in Australian communities. Prepared in a highly accelerated timeframe, this protocol is the product of an effective partnership between multiple stakeholders, which is now a validated and valuable resource at the disposal of the WA Government and community as it continues to navigate the future peaks and troughs of the ongoing COVID-19 pandemic.

## Conclusion

This protocol describes the activities of the DETECT Schools Study. Initiated by the WA state government, this study seeks to understand the extent of asymptomatic transmission of SARS-CoV-2 in schools, to extend the public health contact tracing response to COVID-19 close contacts by determining the SARS-CoV-2 secondary attack rate in school settings, and to investigate the psycho-social wellbeing factors associated with the impacts of the COVID-19 pandemic in school communities.

## Ethics Statement

This study was approved by human ethics review committees at the Child and Adolescent Health Service (PRN RGS0000004059) and the WA Aboriginal Health Ethics Committee (PRN 993), and is conducted in partnership with the WA Departments of Health and Education. Study findings will be shared with stakeholders through regular reporting, and with the academic, medical, and broader communities through scientific publications, presentations, and media releases. Testing numbers and results will be shared iteratively with the public through social media. Written informed consent was obtained from the minor's next of kin for the publication of any potentially identifiable images or data included in this article.

## Author Contributions

HT contributed to design of the study protocol and drafted the manuscript. MM coordinated the conception and design of the protocol and contributed to critical revision of drafts. ME, JM, NM, AW, KL, SA, TB, AL, and MH contributed to design of the study protocol and critical revision of drafts. FM, SZ, PG, JC, DC, and AB contributed to conception and design of the study protocol and critical revision of drafts. LL, MC, PS, and EC contributed to design of the data analysis plan. AM facilitated community and consumer engagement and contributed to evaluation of the study protocol. All authors contributed to the article and approved the submitted version.

## Conflict of Interest

Telethon Kids Institute authors report grants from the Western Australian Department of Health during the conduct of this study. DC and AB report grants from the Western Australian Department of Health outside the submitted work. AB is an employee of the Western Australian Department of Health. The remaining authors declare that the research was conducted in the absence of any commercial or financial relationships that could be construed as a potential conflict of interest.
