# Supplementary material for: DETECT Schools Study Protocol: A Prospective Observational Cohort Surveillance Study Investigating the Impact of COVID-19 in Western Australian Schools
Source: Front Public Health. 2021 Feb 22;9:636921. doi: 10.3389/fpubh.2021.636921 (PMC7937789; doi:10.3389/fpubh.2021.636921)
Supplement: Supplementary file 1 [file Data_Sheet_1.pdf]

DETECT Schools Study:

Understanding the impact of COVID-19 in Western Australian Schools

Supplementary Table 1. Survey items and sources

Note: items are not in order of final presentation in survey instruments, but are listed in groups by domain. Full survey instruments are available on request.

Student Survey: Years 4-12

Domain	Measure	Question stem	Response set	Grade 4-6 survey	Grade 7-12 survey	Source (where applicable)
**1. DEMOGRAPHICS**		What is the name of your school?	[free text]	X	X	
		What Year level are you in?	Year 4<br>Year 5<br>Year 6	X		
		What Year level are you in?	Year 7<br>Year 8<br>Year 9<br>Year 10<br>Year 11<br>Year 12		X	
		What is your gender?	Male<br>Female<br>Other	X	X	
		How many people, including you, usually live in your home?<br>Do not count pets.	2<br>3<br>4<br>5<br>6<br>7<br>8<br>9<br>10 or more	X	X	Speaking Out Survey: Child
		Does your family speak a language other than English at home?	No, English only<br>Yes, we speak<br>____________________<br>(please specify)	X	X	

Domain	Measure	Question stem	Response set	Grade 4-6 survey	Grade 7-12 survey	Source (where applicable)
		Are you Indigenous?	No<br>Yes, Aboriginal<br>Yes, Torres Strait Islander<br>Yes, both Aboriginal and Torres Strait Islander<br>Other	X	X	
		Do you: (please circle ONE NUMBER for each statement)<br>Have your own mobile phone?<br>Have Internet access at home?<br>Have your own device (e.g. computer, laptop, tablet) at home?<br>Share a device (e.g. computer, laptop, tablet) with others at home?<br>Have a private place to study or join in online classes at home?	Yes<br>No	X	X	
**2. HEALTH AND WELLBEING**						

Domain	Measure	Question stem	Response set	Grade 4-6 survey	Grade 7-12 survey	Source (where applicable)
Physical health	Adapted CoRonavIruS Health Impact Survey (CRISIS) V0.3	How would you rate your overall physical health?	Excellent<br>Very good<br>Good<br>Fair<br>Poor	X	X	https://www.nlm.nih.gov/dr2/CRISIS_Adult_Self-Report_Baseline_Current_Form_V0.3.pdf
		In general, how would you say your health is?				Speaking Out Survey_child
Wellbeing and mental health	Child Health Utility 9D (CHU9)	Do you feel worried? *<br>Select the one which is most like you today	I don't feel worried today<br>I feel a little bit worried today<br>I feel a bit worried today<br>I feel quite worried today<br>I feel very worried today		X	© The University of Sheffield 18.01.2008<br><br>Stevens, K. (2012). Valuation of the Child Health Utility 9D Index. *PharmacoEconomics, 30*(8), 729-747. doi:10.2165/11599120-000000000-00000
	Child Health Utility 9D (CHU9)	Do you feel sad? *<br>Select the one which is most like you today	I don't feel sad today<br>I feel a little bit sad today<br>I feel a bit sad today<br>I feel quite sad today<br>I feel very sad today		X	© The University of Sheffield 18.01.2008<br><br>Stevens, K. (2012). Valuation of the Child Health Utility 9D Index. *PharmacoEconomics, 30*(8), 729-747. doi:10.2165/11599120-000000000-00000

Domain	Measure	Question stem	Response set	Grade 4-6 survey	Grade 7-12 survey	Source (where applicable)
	Child Health Utility 9D (CHU9)	Do you feel pain? * Select the one which is most like you today	I don't have any pain today<br>I have a little bit of pain today<br>I have a bit of pain today<br>I have quite a lot of pain today<br>I have a lot of pain today		X	© The University of Sheffield 18.01.2008<br><br>Stevens, K. (2012). Valuation of the Child Health Utility 9D Index. *PharmacoEconomics, 30*(8), 729-747. doi:10.2165/11599120-000000000-00000
	Child Health Utility 9D (CHU9)	Do you feel tired? * Select the one which is most like you today	I don't feel tired today<br>I feel a little bit tired today<br>I feel a bit tired today<br>I feel quite tired today<br>I feel very tired today		X	© The University of Sheffield 18.01.2008<br><br>Stevens, K. (2012). Valuation of the Child Health Utility 9D Index. *PharmacoEconomics, 30*(8), 729-747. doi:10.2165/11599120-000000000-00000
	Child Health Utility 9D (CHU9)	Do you feel annoyed? * Select the one which is most like you today	I don't feel annoyed today<br>I feel a little bit annoyed today<br>I feel a bit annoyed today<br>I feel quite annoyed today<br>I feel very annoyed today		X	© The University of Sheffield 18.01.2008<br><br>Stevens, K. (2012). Valuation of the Child Health Utility 9D Index. *PharmacoEconomics, 30*(8), 729-747. doi:10.2165/11599120-000000000-00000

Domain	Measure	Question stem	Response set	Grade 4-6 survey	Grade 7-12 survey	Source (where applicable)
	Child Health Utility 9D (CHU9)	Do you have problems with school work/ homework (such as reading, writing, doing lessons)? * Select the one which is most like you today	I have no problems with my schoolwork/ homework today<br>I have a few problems with my schoolwork/homework today<br>I have some problems with my schoolwork/homework today<br>I have many problems with my schoolwork/homework today<br>I can't do my schoolwork/homework today		X	© The University of Sheffield 18.01.2008<br><br>Stevens, K. (2012). Valuation of the Child Health Utility 9D Index. *PharmacoEconomics, 30*(8), 729-747. doi:10.2165/11599120-000000000-00000
	Child Health Utility 9D (CHU9)	Do you have problems with sleep? * Select the one which is most like you today	last night I had no problems sleeping<br>last night I had a few problems sleeping<br>last night I had some problems sleeping<br>last night I had many problems sleeping<br>last night I couldn't sleep at all		X	© The University of Sheffield 18.01.2008<br><br>Stevens, K. (2012). Valuation of the Child Health Utility 9D Index. *PharmacoEconomics, 30*(8), 729-747. doi:10.2165/11599120-000000000-00000

Domain	Measure	Question stem	Response set	Grade 4-6 survey	Grade 7-12 survey	Source (where applicable)
	Child Health Utility 9D (CHU9)	Do you have problems with your daily routine (things like eating, having a bath/shower, getting dressed)? * Select the one which is most like you today	I have no problems with my daily routine today<br>I have a few problems with my daily routine today<br>I have some problems with my daily routine today<br>I have many problems with my daily routine today<br>I can't do my daily routine today		X	© The University of Sheffield 18.01.2008<br><br>Stevens, K. (2012). Valuation of the Child Health Utility 9D Index. *PharmacoEconomics, 30*(8), 729-747. doi:10.2165/11599120-000000000-00000
	Child Health Utility 9D (CHU9)	Are you able to join in activities (things like being with your friends, joining in things)? * Select the one which is most like you today	I can join in with any activities today<br>I can join in with most activities today<br>I can join in with some activities today<br>I can join in with a few activities today<br>I can join in with no activities today		X	© The University of Sheffield 18.01.2008<br><br>Stevens, K. (2012). Valuation of the Child Health Utility 9D Index. *PharmacoEconomics, 30*(8), 729-747. doi:10.2165/11599120-000000000-00000
	Overall wellbeing: Children's Worlds – Student Life Satisfaction Scale (SLSS)	How much do you agree or disagree with each of these sentences:<br>My life is going well<br>My life is just right<br>I wish I had a	Strongly agree<br>Agree<br>Neither agree nor disagree<br>Disagree<br>Strongly disagree<br>Don't know	X	X	Scale is not copyrighted and can be used without charge or permission by interested researchers.<br><br>Huebner, E.S. The Students' Life Satisfaction Scale: An assessment of psychometric properties with black and white elementary school students. *Soc Indic Res* 34, 315–323 (1995).

Domain	Measure	Question stem	Response set	Grade 4-6 survey	Grade 7-12 survey	Source (where applicable)
		different kind of life<br>I have a good life<br>I have what I want in life<br>I feel positive about my future				https://doi.org/10.1007/BF01078690
Relationships	National Survey of Child and Adolescent Mental Health and Well being 2012	About how many friends do you have who you either hang out with, talk to on the phone, regularly send messages to, either through social media, chat, gaming or ways online, or get together with socially?<br>(Don't include online friends if you don't talk or message each other regularly)	0 Friends<br>1 Friend<br>2 Friends<br>3 Friends<br>4 Friends<br>5 Friends<br>6 – 10 Friends<br>11 – 15 Friends<br>15 – 20 Friends<br>20 Friends or more	X	X	
	National Survey of Child and Adolescent Mental Health and Well being 2012	How much can you rely on your friends for help if you have a serious problem?	A lot<br>Some<br>A little<br>Not at all	X	X	

Domain	Measure	Question stem	Response set	Grade 4-6 survey	Grade 7-12 survey	Source (where applicable)
School enjoyment	National Survey of Child and Adolescent Mental Health and Well being 2012	How do you feel about going to school?*	I like school very much<br>I like school quite a bit<br>I neither like nor dislike school<br>I don't like school very much<br>I hate school		X	
	ACER School Life questionnaire	For each sentence, circle the number that shows how much you agree or disagree.<br>My school is a place where:<br>I feel happy<br>I really like to go to each day<br>I find that learning is a lot of fun<br>I feel safe and secure<br>I like learning<br>I get enjoyment from being there	Strongly agree<br>Agree<br>Neither agree nor disagree<br>Disagree<br>Strongly disagree	X	X	These were used most recently in the *Second Australian Child and Adolescent Survey of Mental Health and Wellbeing* conducted in 2013-14<br><br>Ainley, J. & Bourke, S. (1992). Students' views of primary school. *Research Papers in Education*, 7(2), 107-128.<br><br>Ainley, J., Reed, R. and Miller, H. (1986). *School Organization and the Quality of Schooling: A Study of Victorian Government Secondary Schools*. (ACER Research Monograph No. 29). Hawthorn, Vic.: ACER.
**3. COVID IMPACT**						

Domain	Measure	Question stem	Response set	Grade 4-6 survey	Grade 7-12 survey	Source (where applicable)
Schooling impact	(adapted)<br>WA Speaking Out Survey_child	If you missed school due to COVID-19, did it worry you?	No<br>Yes, a little<br>Yes, lots	X	X	(adapted)<br>WA Speaking Out Survey_child
Wellbeing impact	(adapted)<br>Measuring Worldwide COVID-19 Attitudes and Beliefs	In the past two weeks how often have you felt the following when you think about COVID-19?<br>Nervous<br>Calm and relaxed<br>Worried about my health<br>Worried about the health of my family members<br>Stressed about leaving my house<br>Lonely<br>Missed seeing my friends	Often<br>Sometimes<br>Never	X	X	Fetzer, T., Witte, M., Hensel, L., Jachimowicz, J.M., Haushofer, J., Ivchenko, A., Caria, C., Reutskaja, E., Roth, C., Fiorin, F., Gomez, M., Kraft-Todd, G., Goetz, F., & Yoeli, E.. Global Behaviors and Perceptions in the COVID-19 Pandemic.<br>https://doi.org/10.31234/osf.io/3kfmh

Domain	Measure	Question stem	Response set	Grade 4-6 survey	Grade 7-12 survey	Source (where applicable)
	COVID-19 Adolescent Symptom & Psychological Experience Questionnaire (CASPE)	Overall, how much has COVID-19 and the resulting changes to daily life affected your life in a good way?<br>(Yr 4-6: Overall, how much has COVID-19 affected your life in a good way?)	Not at all<br>A little<br>Somewhat<br>A lot<br>A great deal	X	X	**Ladouceur, CD**. (2020). COVID-19 Adolescent Symptom & Psychological Experience Questionnaire.
	COVID-19 Adolescent Symptom & Psychological Experience Questionnaire (CASPE)	Overall, how much has COVID-19 and the resulting changes to daily life affected your life in a bad way?<br>(Yr 4-6: Overall, how much has COVID-19 affected your life in a bad way?)	No, not at all<br>A little<br>Somewhat<br>A lot<br>A great deal	X	X	**Ladouceur, CD**. (2020). COVID-19 Adolescent Symptom & Psychological Experience Questionnaire.
	COVID-19 Adolescent Symptom & Psychological Experience Questionnaire (CASPE)	Compared to before the COVID-19 outbreak, how much more have you felt this way in the past 7 days, including today?<br>Relaxed<br>Hopeful<br>Confident about the	Not at all<br>A little<br>Some<br>A lot<br>A great deal	X	X	**Ladouceur, CD**. (2020). COVID-19 Adolescent Symptom & Psychological Experience Questionnaire.

Domain	Measure	Question stem	Response set	Grade 4-6 survey	Grade 7-12 survey	Source (where applicable)
		future<br>Hopeless<br>Anxious/stressed<br>Cheerful				
**4. TESTING**						
		Did you participate in COVID-19 testing (throat and nasal swabs) at your school?	Yes<br>No (skip)<br>Don't know	X	X	
		How concerned were you about being tested for COVID-19 at school?	Not at all concerned<br>A little bit concerned<br>Moderately concerned<br>Quite a bit concerned<br>Very concerned	X	X	
		Did the staff using personal protective equipment (gown, mask, gloves) cause you any concern?	Not at all concerned<br>A little bit concerned<br>Moderately concerned<br>Quite a bit concerned<br>Very concerned	X	X	

Domain	Measure	Question stem	Response set	Grade 4-6 survey	Grade 7-12 survey	Source (where applicable)
		Please rate your discomfort with the swab test	No discomfort<br>Mild discomfort – a tickle in my throat and nose<br>Moderate discomfort – I coughed or gagged or sneezed and didn't like the feeling<br>Painful – but the pain stopped quickly and I would have the swab again<br>Very painful - it took a while to recover and I would not have the swab done again	X	X	
		How important is it to you that your school is participating in this research study COVID-19?	Very important<br>Important<br>Not important<br>Unsure	X	X	

Parent K-12 survey

<table>
<tr><th>Domain</th><th>Measure</th><th>Question stem</th><th>Response set</th><th>Grade K-6 parent survey</th><th>Grade 7-12 parent survey</th><th>Source (where applicable)</th></tr>
<tr><td>1. DEMOGRAPHICS</td><td></td><td></td><td></td><td></td><td></td><td></td></tr>
<tr><td rowspan="3">Child Demographics</td><td></td><td>For how many children (under age 18) are you the parent/carer?</td><td>One
Two
Three
Four
Five or more</td><td>X</td><td>X</td><td></td></tr>
<tr><td></td><td>What is the age and gender of the primary* secondary school aged children in your care?</td><td><table><tr><th>Child</th><th>Age (yrs)</th><th>Gender</th></tr><tr><td>1</td><td></td><td></td></tr><tr><td>2</td><td></td><td></td></tr><tr><td>3</td><td></td><td></td></tr><tr><td>4</td><td></td><td></td></tr><tr><td>5</td><td></td><td></td></tr></table></td><td>X</td><td>X</td><td></td></tr>
<tr><td></td><td>What primary school does your youngest /oldest child attend?</td><td>[free text]</td><td>X</td><td></td><td></td></tr>
</table>

Domain	Measure	Question stem	Response set	Grade K-6 parent survey	Grade 7-12 parent survey	Source (where applicable)
		What secondary school does your youngest /oldest child attend?	[free text]		X	
		What is your relationship to your youngest /oldest child?	Mother / Stepmother<br>Father / Stepfather<br>Grandmother<br>Grandfather<br>Other	X	X	
		In what primary year is your youngest /oldest child currently enrolled?	Kindergarten<br>Pre Primary<br>Year 1<br>Year 2<br>Year 3<br>Year 4<br>Year 5<br>Year 6	X		
		What secondary school year is your child currently enrolled in?	Year 7<br>Year 8<br>Year 9<br>Year 10<br>Year 11<br>Year 12		X	

Domain	Measure	Question stem	Response set	Grade K-6 parent survey	Grade 7-12 parent survey	Source (where applicable)
		What type of family setting does your youngest /oldest child live for most days each week?	Two parent family<br>Single parent family<br>Step / blended family<br>Other (please indicate)<br>______________________	X	X	
		Does your youngest /oldest child have a health problem / disability that may put them at greater risk of contracting COVID-19?	No<br>Yes<br>Maybe<br>Don't know	X	X	
Parent demographics		What is your gender?	Male<br>Female<br>Other	X	X	
		What is your age?	Younger than 35 years<br>35 – 44 years<br>45 – 54 years<br>55 – 64 years<br>65 years and older	X	X	
		What is your country of birth?	Australia<br>Other<br>Please specify:<br>______________	X	X	

Domain	Measure	Question stem	Response set	Grade K-6 parent survey	Grade 7-12 parent survey	Source (where applicable)
		Do you speak a language other than English at home?	No<br>Yes - please specify<br>________________	X	X	
		Are you Indigenous?	No<br>Yes, Aboriginal<br>Yes, Torres Strait Islander<br>Yes, both Aboriginal and Torres Strait Islander<br>Other	X	X	
**2. HEALTH AND WELLBEING**						
Adult: Wellbeing and mental health	WHO (Five) Well-Being Index (1998 version)	Over the last two weeks:<br>I have felt cheerful and in good spirits<br>I have felt calm and relaxed<br>I have felt active and vigorous<br>I woke up feeling fresh and rested<br>My daily life has been filled with things that interest me<br>I have felt safe	All the time<br>Most of the time<br>More than half of the time<br>Less than half of the time<br>Some of the time<br>At no time	X	X	WHO. (1998). Wellbeing Measures in Primary Health Care/The Depcare Project. WHO Regional Office for Europe: Copenhagen.

Domain	Measure	Question stem	Response set	Grade K-6 parent survey	Grade 7-12 parent survey	Source (where applicable)
	ABS (General Social Survey) – Overall Life Satisfaction	All things considered, how satisfied are you with your life? Again, pick a number between 0 and 10 to indicate how satisfied you are.	1-10<br>Refused<br>Don't know	X	X	
	1991 WA Child Health Survey	Have you felt strain, stress or pressure during the past 6 months?	Yes, almost more than I can take<br>Yes, quite a bit of pressure<br>Yes, some/more than usual<br>Yes, a little/about usual<br>Not at all	X	X	
Child: Wellbeing and mental health	Adapted CoRonavIruS Health Impact Survey (CRISIS) V0.3	Over the past 2 weeks, how worried was your child generally? *	Not worried at all<br>Slightly worried<br>Moderately worried<br>Very worried<br>Extremely worried	X		https://www.nlm.nih.gov/dr2/CRISIS_Adult_Self-Report_Baseline_Current_Form_V0.3.pdf

Domain	Measure	Question stem	Response set	Grade K-6 parent survey	Grade 7-12 parent survey	Source (where applicable)
	Adapted CoRonavIruS Health Impact Survey (CRISIS) V0.3	Over the past 2 weeks, how happy versus sad was your child? *	Very sad/depressed/unhappy<br>Moderately sad/depressed/unhappy<br>Neutral<br>Moderately happy/cheerful<br>Very happy/cheerful;	X		https://www.nlm.nih.gov/dr2/CRISIS_Adult_Self-Report_Baseline_Current_Form_V0.3.pdf
	Adapted CoRonavIruS Health Impact Survey (CRISIS) V0.3	Over the past 2 weeks, how much has your child been able to enjoy his/her usual activities? *	Not at all<br>Slightly<br>Moderately<br>Very much<br>A lot	X		https://www.nlm.nih.gov/dr2/CRISIS_Adult_Self-Report_Baseline_Current_Form_V0.3.pdf
	Adapted CoRonavIruS Health Impact Survey (CRISIS) V0.3	Over the past 2 weeks, how relaxed versus anxious was your child? *	Very relaxed/calm<br>Moderately relaxed/calm<br>Neutral relaxed/calm<br>Moderately nervous/anxious<br>Very nervous anxious	X		https://www.nlm.nih.gov/dr2/CRISIS_Adult_Self-Report_Baseline_Current_Form_V0.3.pdf

Domain	Measure	Question stem	Response set	Grade K-6 parent survey	Grade 7-12 parent survey	Source (where applicable)
	Adapted CoRonavIruS Health Impact Survey (CRISIS) V0.3	Over the past 2 weeks, how fidgety or restless was your child? *	Not restless at all<br>Slightly restless<br>Moderately restless<br>Very restless<br>Extremely restless	X		https://www.nlm.nih.gov/dr2/CRISIS_Adult_Self-Report_Baseline_Current_Form_V0.3.pdf
	Adapted CoRonavIruS Health Impact Survey (CRISIS) V0.3	Over the past 2 weeks, how fatigued or tired was your child? *	Not fatigued or tired at all<br>Slightly fatigued or tired<br>Moderately fatigued or tired<br>Very fatigued or tired<br>Extremely fatigued or tired	X		https://www.nlm.nih.gov/dr2/CRISIS_Adult_Self-Report_Baseline_Current_Form_V0.3.pdf
	Adapted CoRonavIruS Health Impact Survey (CRISIS) V0.3	Over the past 2 weeks, for their age, how well has your child been able to concentrate or focus? *	Very focused/attentive<br>Moderately focused/attentive<br>Neutral<br>Moderately unfocused/distracted<br>Very unfocused/distracted	X		https://www.nlm.nih.gov/dr2/CRISIS_Adult_Self-Report_Baseline_Current_Form_V0.3.pdf

Domain	Measure	Question stem	Response set	Grade K-6 parent survey	Grade 7-12 parent survey	Source (where applicable)
	Adapted CoRonavIruS Health Impact Survey (CRISIS) V0.3	Over the past 2 weeks, how irritable or easily angered has your child been? *	Not irritable or easily angered<br>Slightly irritable or easily angered<br>Moderately irritable or easily angered<br>Very irritable or easily angered<br>Extremely irritable or easily angered	X		https://www.nlm.nih.gov/dr2/CRISIS_Adult_Self-Report_Baseline_Current_Form_V0.3.pdf
	Adapted CoRonavIruS Health Impact Survey (CRISIS) V0.3	Over the past 2 weeks, how lonely has your child been? *	Not lonely at all<br>Slightly lonely<br>Moderately lonely<br>Very lonely<br>Extremely lonely	X		https://www.nlm.nih.gov/dr2/CRISIS_Adult_Self-Report_Baseline_Current_Form_V0.3.pdf
Sleep	Adapted CoRonavIruS Health Impact Survey (CRISIS) V0.3	Over the past 2 weeks, how many hours per night did your child sleep on average? *	Numeric	X		https://www.nlm.nih.gov/dr2/CRISIS_Adult_Self-Report_Baseline_Current_Form_V0.3.pdf

Domain	Measure	Question stem	Response set	Grade K-6 parent survey	Grade 7-12 parent survey	Source (where applicable)
Physical health	Adapted CoRonavIruS Health Impact Survey (CRISIS) V0.3	How would you rate your child's overall physical health?	Excellent<br>Very good<br>Good<br>Fair<br>Poor	X		https://www.nlm.nih.gov/dr2/CRISIS_Adult_Self-Report_Baseline_Current_Form_V0.3.pdf
**3. IMPACT OF COVID**						
Schooling impact		When students were permitted to learn at home in Weeks 1 to 3 of Term 2 this year, did your family choose to:	Keep your child at home<br>Send your child to school<br>A mixture of both	X	X	

Domain	Measure	Question stem	Response set	Grade K-6 parent survey	Grade 7-12 parent survey	Source (where applicable)
		If you chose to keep your child at home, what were your reasons?<br>(Please mark all that apply)	I was told to do so by the Government<br>I was told to do so by my child's school<br>To keep my child and my family safe from COVID-19<br>To keep the school community safe from COVID-19<br>To keep the greater community safe from COVID-19<br>My child was not coping with the COVID-19 situation<br>Members of my family were not coping with the COVID-19 situation<br>My decision was influenced by my family's financial circumstances<br>Other: ______________________	X	X	

Domain	Measure	Question stem	Response set	Grade K-6 parent survey	Grade 7-12 parent survey	Source (where applicable)
		If you chose to keep your child at school, what were your reasons?<br>(Please mark all that apply)	I felt it was safe to do so<br>My child would receive a better education at school<br>I needed to work<br>I did not feel comfortable supporting learning at home<br>My family's financial circumstances.<br>There were home factors that meant it was better for my child to be at school<br>My child missed spending time with his/her school friends.<br>Other (please specify): ________________________	X	X	
		Has your child returned to their school (on school grounds) full-time?	Yes<br>No		X	

Domain	Measure	Question stem	Response set	Grade K-6 parent survey	Grade 7-12 parent survey	Source (where applicable)
		Considering the past 4 weeks, possibly including learning at home, how much do you agree or disagree with the following statements?<br>I felt informed by my child's school regarding how the school was responding to COVID-19<br>I felt my child was safe at school<br>I was worried my child was missing essential learning because of COVID-19<br>Overall, my current experience with my child's school's response to COVID-19 was positive	Strongly agree<br>Agree<br>Neither agree nor disagree<br>Disagree<br>Strongly disagree<br>Not applicable	X	X	

Domain	Measure	Question stem	Response set	Grade K-6 parent survey	Grade 7-12 parent survey	Source (where applicable)
Resources		To what extent did you have the following for your child when learning at home during the COVID-19 situation?<br>Computing resources such as desktop or laptop computers to complete schooling online<br>Time to assist my child with learning at home tasks<br>Reliable internet connection<br>Resources provided by the school for learning at home<br>Enough appropriate places for my child to study, such as a quiet space with a table/desk	Always<br>Most of the time<br>Sometimes<br>Rarely<br>Never	X	X	

Domain	Measure	Question stem	Response set	Grade K-6 parent survey	Grade 7-12 parent survey	Source (where applicable)
Parent self efficacy – child's resilience	Resilience Scale	Please indicate how much you agree or disagree with each of the following statements.<br>I know how to build resilience in my child to help them deal with stress<br>I have the skills to help my child to cope with change<br>I know how to help my child to take action if they feel isolated/lonely<br>I am able to help my child if they are feeling anxious or sad	Strongly agree<br>Agree<br>Neither agree nor disagree<br>Disagree<br>Strongly disagree	X	X	
Parent wellbeing	COVID-19 Adolescent Symptom & Psychological Experience Questionnair Questionnair	Compared to before the COVID-19 outbreak, how much more have you felt this way in the past 7 days, including today?	Not at all<br>A little<br>Some<br>A lot<br>A great deal	X	X	Ladouceur, CD. (2020). COVID-19 Adolescent Symptom & Psychological Experience Questionnaire.

Domain	Measure	Question stem	Response set	Grade K-6 parent survey	Grade 7-12 parent survey	Source (where applicable)
	e (CASPE)	Relaxed<br>Hopeful<br>Confident about the future<br>Hopeless<br>Anxious/stressed<br>Cheerful				
Other impact		Was your family impacted in other ways by having your child learn from home? If so, how?	[free text]	X	X	
**4. TESTING**						
		Did your child participate in the COVID-19 testing (throat and nasal swabs) at school as part of the DETECT study?	Yes<br>No<br>Don't Know	X	X	
		On the morning of the COVID testing how concerned did your child feel about possibly	Not at all concerned<br>A little bit concerned<br>Moderately concerned<br>Quite a bit concerned	X	X	

Domain	Measure	Question stem	Response set	Grade K-6 parent survey	Grade 7-12 parent survey	Source (where applicable)
		having their throat and nose swabbed at school?	Extremely concerned			
		If your child was swabbed how did he/she feel after having their throat and nose swabbed at school?	Not at all concerned<br>A little bit concerned<br>Moderately concerned<br>Quite a bit concerned<br>Extremely concerned	X	X	
		How concerned are/were you about the results of the swab testing for your child?	Not at all concerned<br>A little bit concerned<br>Moderately concerned<br>Quite a bit concerned<br>Extremely concerned	X	X	
		How important is it to you that your child's school is participating in this research study about COVID-19?	Very important<br>Important<br>Not important<br>Unsure	X	X	
		Please write below any other comments you have about what could be done to support children, schools and families currently.	[free text]	X	X	

School staff survey

Domain	Measure	Question stem	Response set	Source (where applicable)
**1. DEMOGRAPHICS**				
School information		Which position best describes your role in your school?	Classroom teacher<br>Classroom-based support staff (e.g. education assistant) [skip to q8]<br>Administrative staff (Principal, deputy principal, school officer) [skip to q8]<br>Other support staff (psychologist, nurse, cleaner, grounds person) [skip to q8]	
		In which year cluster/s do you teach this year?	Kindergarten/pre-primary<br>Years 1-3<br>Years 4-6<br>Years 7-10<br>Years 11-12	
		In which learning area/s do you teach this year?	All of the following<br>The Arts<br>English<br>Health and Physical Education<br>Humanities and Social Sciences<br>Science<br>Maths<br>Languages other than English<br>Technology and Enterprise<br>Other __________(please specify)	

Domain	Measure	Question stem	Response set	Source (where applicable)
		What type of school do you work in? [One option only]	Education support school or centre<br>Primary school<br>Secondary school<br>Combined primary/secondary school<br>Residential college	
		My school is in a:	North or South metropolitan education region<br>Non-metropolitan education region	
Personal information		What is your gender?	Male<br>Female<br>Other	
		What is your age?	Younger than 35 years<br>35 – 44 years<br>45 – 54 years<br>55 – 64 years<br>65 years and older	
**2. HEALTH AND WELLBEING**				
	WHO (Five) Well-Being Index (1998 version)	Over the last two weeks:<br>I have felt cheerful and in good spirits<br>I have felt calm and relaxed<br>I have felt active and vigorous<br>I woke up feeling fresh and re-sted<br>My daily life has been filled with	All of the time<br>Most of the time<br>More than half of the time<br>Less than half of the time<br>Some of the time<br>At no time	WHO. (1998). Wellbeing Measures in Primary Health Care/The Depcare Project. WHO Regional Office for Europe: Copenhagen.

Domain	Measure	Question stem	Response set	Source (where applicable)
		things that interest me		
	ABS (General Social Survey) – Overall Life Satisfaction	All things considered, how satisfied are you with your life? Again, pick a number between 0 and 10 to indicate how satisfied you are.	0-10	
	1991 WA Child Health Survey	Have you felt strain, stress or pressure during the past 6 months?	Yes, almost more than I can take<br>Yes, quite a bit of pressure<br>Yes, some/more than usual<br>Yes, a little/about usual<br>Not at all	
**3. COVID IMPACT**				
Wellbeing impact		Compared to before the COVID-19 outbreak, how much more have you felt this way in the past 7 days, including today?<br>Relaxed<br>Hopeful<br>Confident about the future<br>Hopeless<br>Anxious /stressed<br>Cheerful	Not at all<br>A little<br>Some<br>A lot<br>A great deal	
School/employment impact		Considering how you have been feeling in response to the COVID-19 pandemic, how much do you agree or disagree with the	Strongly Agree<br>Agree<br>Neither agree nor disagree<br>Disagree	

Domain	Measure	Question stem	Response set	Source (where applicable)
		following statements?<br>I feel my school has prepared a safe and protected working environment<br>I am able to safely physical distance when at school<br>I am provided with enough current information to feel secure in the workplace<br>I am concerned about going to work due to my potential COVID-19 risk<br>I have adequate access to workplace support services<br>I have adequate support from others to cope with the changes relevant to my role<br>I am concerned about students who are not coping or who have educational needs greater than I can support	Strongly disagree	
		To what extent has the COVID-19 situation:<br>Reduced opportunities to meet the learning needs of students?<br>Provided new ways to engage with parents?	A lot<br>Somewhat<br>A little<br>No different	

Domain	Measure	Question stem	Response set	Source (where applicable)
		Increased your workload?<br>Improved your IT capabilities?<br>Reduced the engagement of students?<br>Increased the amount of time spent supervising students (e.g. duty time, movement around the school, use of equipment)?<br>Negatively impacted student wellbeing?<br>Negatively impacted student attendance?		
		Do you feel you need more support or advice currently in relation to the following areas?<br>Modifying the curriculum to cover gaps in student learning caused by the COVID-19 situation*<br>Appropriate ways to teach students how to think about/cope with COVID-19*<br>Teaching safe hygiene practices to students*<br>Teaching students online and digital technology*<br>How to help parents oversee their children's learning at home.*	Yes<br>No	

Domain	Measure	Question stem	Response set	Source (where applicable)
		Work life balance and supporting my own wellbeing to cope with COVID-19<br>Supporting my colleagues' wellbeing<br>Please describe below any other areas not listed where you currently need support or advice:<br>____________________		
**4. TESTING**				
		Did you participate in the COVID-19 testing (throat and nasal swabs) at school as part of the DETECT study?	Yes<br>No<br>Don't Know	
		Please rate your discomfort with the swab test	No discomfort<br>Mild discomfort – a tickle in my throat and nose<br>Moderate discomfort – I coughed or gagged or sneezed and didn't like the feeling<br>Painful - but the pain stopped quickly and I would have the swab done again<br>Very painful - It took a while to recover and I would not have the swab done again	

Domain	Measure	Question stem	Response set	Source (where applicable)
		How concerned are you about the results from the swab test?	Not at all concerned<br>A little bit concerned<br>Moderately concerned<br>Quite a bit concerned<br>Extremely concerned	
		How important is it to you that your school is participating in this research about COVID-19?	Very important<br>Important<br>Not important<br>Unsure	
		In your opinion, how disruptive was the COVID-19 testing of the staff and students to the school?	Not at all<br>A little bit<br>Moderately<br>Quite a bit<br>Extremely	
		Please write below any comments you have about what could be done to support children, schools and families currently.	[free text]	
